# Use of Artificial Neural Networks to Examine Parameters Affecting the Immobilization of Streptokinase in Chitosan 

**Published:** 2014

**Authors:** Seyed Mohamad Sadegh Modaresi, Mohammad Ali Faramarzi, Arash Soltani, Hadi Baharifar, Amir Amani

**Affiliations:** a *Departemant of Biology, Faculty of Basic Sciences, Kharazmi University, Tehran, Iran. *; b *Biotechnology Research Center, School of Pharmacy, Tehran University of Medical Sciences, Tehran, Iran.*; c * Department of Pharmaceutical Biotechnology, School of Pharmacy, Tehran University of Medical Sciences, Tehran, Iran.*; d *Department of Medical Nanotechnology, School of Advanced Technologies in Medicine, Tehran University of Medical Sciences, Tehran, Iran.*; e *Medical Biomaterials Research Center, Tehran University of Medical Sciences, Tehran, Iran.*

**Keywords:** Artificial neural networks, Streptokinase, Chitosan, Half-life, Electrostatic interactions

## Abstract

Streptokinase is a potent fibrinolytic agent which is widely used in treatment of deep vein thrombosis (DVT), pulmonary embolism (PE) and acute myocardial infarction (MI). Major limitation of this enzyme is its short biological half-life in the blood stream. Our previous report showed that complexing streptokinase with chitosan could be a solution to overcome this limitation. The aim of this research was to establish an artificial neural networks (ANNs) model for identifying main factors influencing the loading efficiency of streptokinase, as an essential parameter determining efficacy of the enzyme. Three variables, namely, chitosan concentration, buffer pH and enzyme concentration were considered as input values and the loading efficiency was used as output. Subsequently, the experimental data were modeled and the model was validated against a set of unseen data. The developed model indicated chitosan concentration as probably the most important factor, having reverse effect on the loading efficiency.

## Introduction

Immobilization of enzymes have been performed by different methods which are generally classified into two categories: immobilizations using weak interactions between support and enzyme (*e.g*. electrostatic interactions) and those that are based on binding of enzymes to a support by means of covalent bonds (1, 2). Several advantages have been reported for using weak interactions over covalent bindings. First, this technique is inexpensive and involves a mild and simple process. In addition, no pre-treatment on enzyme or support is generally required, and consequently, the conformation of the protein is not strongly influenced by the process. Finally, the mentioned strategy avoids the use of reagents causing possible toxicities and other undesirable effects (1). 

Reviewing the literature shows that when using complexation between oppositely charged macromolecules, loading efficiency of the enzyme depends on many different factors. Xu and Du (3) produced various formulations of chitosan/TPP nanoparticles to deliver bovine serum albumin (BSA). They examined the effects of factors such as molecular weight and deacetylation degree of chitosan, as well as concentration of chitosan and BSA. The first two factors showed a direct relation with encapsulation efficiency of BSA, while an increase in concentration of chitosan or BSA led to a decrease in loading efficiency. The increase in concentration of ammonium glycyrrhizinate and chitosan was shown to decrease the encapsulation efficiency (4). Zhang *et al*. (5) used water soluble chitosan/TPP nanoparticles as carrier for BSA and reported that the encapsulation efficiency decreased from 95 to 40% by increasing the BSA concentration from 0.1 to 2.0 mg/mL. Similarly, loading efficiency was reported to decrease from about 60% to 30% when BSA concentration increased in an alginate coated BSA-loaded chitosan system (6). This trend is however to some extent contradictory in the literature and does not appear to be generalisable. For instance, the encapsulation efficiency in a BSA-loaded chitosan-TPP system, increased from 38.7% to 72.5% with an increase in BSA concentration from 0.25 to 1.5 mg/mL (7). A similar finding has been reported by Zhang *et al.* (8) where BSA was loaded into the chitosan nanoparticles. 

To summarize the above mentioned works, no one appears to be comprehensive; rather, in general, they have employed one-factor-at-a-time approaches. This approach is commonly associated with difficulties such as incorrect estimates in the effects of independent factors, incomplete coverage of factor space as well as lack of systematic assessments for interactions between independent/dependent factors (9).

Streptokinase is highly immunogenic, thus, rapidly cleared from the plasma, ending-up in short biological half-life. To overcome this, various carriers such as polymers and liposomes have been used for encapsulating streptokinase in therapeutic applications (-). In our previous report, *β*-hemolytic extracellular enzyme streptokinase was loaded on chitosan and an improved biological half-life was obtained for the loaded enzyme (13). The streptokinase incorporated in chitosan nanoparticles was shown to remain stable in the body with a prolonged *in-vivo* half-life compared to naked enzyme, indicating the potential of electrostatic interactions in preparing streptokinase-loaded chitosan nanoparticles. 

In this study, hypothesizing that the half-life of the enzyme greatly depends on its loading efficiency (14, 15), parameters that potentially affect the loading efficiency of streptokinase on the chitosan have been investigated using artificial neural networks (ANNs). ANNs are usually used to model complex relationships between inputs and outputs or to find patterns in data, where standard statistical analyses often fail to work (16, 17), as commonly observed in nanotechnology experiments (18). In this work, from different variables affecting the streptokinase loading efficiency, three parameters, namely, chitosan concentration, buffer pH and enzyme concentration were picked to be analyzed and model their effects on the loading efficiency of streptokinase using ANNs.

## Experimental

*Materials and methods*

Pure recombinant streptokinase was supplied by Pasteur institute of Iran (Tehran, Iran), chitosan (average molecular weight ~ 500kDa, DD~93%) was purchased from Easter Holding Group (China) and acetate buffer solution was obtained from Merck chemicals co. (Germany).

*Preparation of streptokinase-loaded chitosan nanoparticles*

27 samples having different chitosan and streptokinase concentrations (*i.e*. 0.1 to 1 mg/mL and 0.1 to 0.4 mg/mL, respectively) were prepared in acetate buffer with pH values between 4.83 and 6.30, as detailed in Tables 1 and 2. The method of preparation comprised of dropwise addition of streptokinase to the chitosan solution, followed by a 60 min stirring at 4 ˚C. The nanosuspension was then centrifuged 12000×g for 40 min at 4 ºC. Afterwards, the collected supernatant was utilized for calculating the enzyme entrapment efficiency by Bradford protein assay technique (13). The data were then employed to evaluate the impact of the variables on the loading efficiency of enzyme with regards to the model obtained from ANNs.

**Table 1:** The training and tests data sets used in ANNs modelling

**Chitosan Concentration** **(mg/mL)**	**pH**	**Enzyme Concentration** **(mg/mL)**	**Loading Scale** **(%)**	**Predicted (%)**	**Error (%)**
0.50	5.73	0.10	33	27.9	-5.1
1.00	5.85	0.20	21	21.6	0.6
0.75	5.97	0.40	19	19.0	0.0
1.00	6.30	0.20	11	12.6	1.6
0.30	4.83	0.20	34	31.9	-2.1
0.65	5.22	0.10	27	38.2	11.2
0.33	5.30	0.10	48	44.9	-3.1
0.20	5.57	0.10	49	43.2	-5.8
0.16	5.65	0.10	45	47.6	2.6
0.25	5.80	0.10	25	33.1	8.1
0.20	5.90	0.10	59	53.9	-5.1
0.18	6.00	0.20	70	69.6	-0.4
0.45	5.10	0.10	49	38.1	-10.9
0.15	4.90	0.10	29	36.9	7.9
0.50	4.97	0.20	37	32.9	-4.1
0.10	4.90	0.30	43	43.9	0.9
0.70	5.10	0.20	35	33.5	-1.5
0.43	4.85	0.25	21	30.5	9.5
0.50	4.90	0.30	36	30.9	-5.1
0.31	5.22	0.20	52	43.9	-8.1*
0.85	6.20	0.10	15	13.2	-1.8*

* The last 2 data show the test data.

**Table 2:** The validation (unseen) data sets used in ANNs modelling

**Chitosan Concentration** **(Mg/mL)**	**pH**	**Enzyme Concentration** **(Mg/mL)**	**Loading Scale** **(%)**	**Predicted (%)**	**Error (%)**
0.38	5.40	0.1	50	44.2	-5.8
1.00	6.12	0.3	14	13.4	-0.6
0.25	5.45	0.1	33	44.8	11.8
0.78	5.92	0.2	18	17.3	-0.7
0.75	4.88	0.1	25	27.6	2.6
0.50	5.46	0.1	42	42.3	0.3

*Artificial neural networks studies*

Relations between inputs and the output were modelled using INForm v4.02 (Intelligensys, UK), as a commercial ANNs software. As mentioned above, three factors, namely, chitosan concentration (mg/mL), enzyme concentration (mg/mL) and buffer pH were considered as input variables and the output variable was loading efficiency of streptokinase (%). The network was trained using the data listed in Table 1 and the training parameters listed in Table 3. Subsequent to training procedure, the developed model was validated against the set of unseen data listed in Table 2. Afterwards, the response surfaces, generated from the model, were employed to obtain understanding about the rules governing the relations between the inputs and the output, as detailed previously (19, 20). As discussed above, the data were split into three sets: the training data set train the network and achieve the relations between the inputs/output variables, the test data to prevent overtraining (see Table 1) and the unseen (validation) data to evaluate the predictability of the obtained model (see Table 2). Further details of the modelling procedure have been given previously (21).

**Table 3:** The training parameters set with INForm v4.02.

**Network structure**	No. of hidden layers	1
	No. of nodes in hidden layer	3
**Backpropagation type**		Incremental
**Transfer function**	Output	Tanh
	Hidden layer	Symmetric Sigmoid

## Results and Discussion

The design of proper nanocarriers for drug delivery purposes is a major area of research in nanomedicine. The most common carriers for drug delivery include polymeric ones where drugs are loaded onto biodegradable, non-toxic polymer-based supports. Our work consisted of loading streptokinase as an important and efficient fibrinolytic agent onto chitosan, a non-toxic, biodegradable and economically ideal polymer through electrostatic interactions. ANNs were then used to optimize our previously proposed nano-system containing streptokinase (13). ANNs are based on a working brain with interconnection and arrangement of neurons in different layers to create networks, where learning results in network function (22). Compared with classic modeling techniques such as response surface methodology (RSM), ANNs have shown promising in terms of their estimation and prediction capabilities (23, 24). Additionally, ANNs have been proved to be able in dealing with nonlinear relations which are commonly observed in nano-based products, where statistical approaches normally fail to work, a second reason for using ANNs in this study (18).

The results showed a coefficient of determination (R^2^) of 0.85 for unseen data which represents a desirable predictive ANNs model. This model was then used to study the influence of the three different input variables on the streptokinase loading efficiency. To understand the effects of different factors on the output in an ANNs model, use of sensitivity analysis is the first choice someone can make. In this study, to investigate the relationships between inputs and output we used response surfaces, as detailed previously (21, 25). To summarize the method, this strategy examines the influence of two variables on the output through 3D graphs (*i.e*. response surfaces) generated by the software while the other variable(s) is fixed at low, medium and high values. 

**Figure 1:**
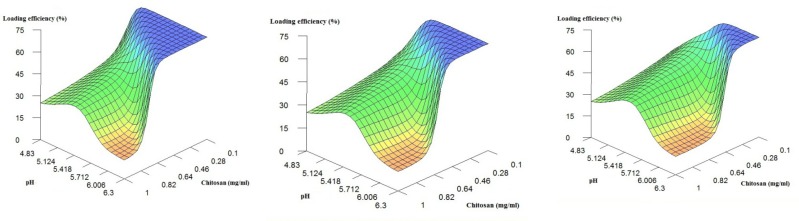
3D Plots of loading efficiency predicted by the ANNs model fixed at low, medium and high concentrations of the enzyme

To do so, we first examined the influence of chitosan concentration and pH on the level of streptokinase loading while the enzyme concentration is fixed at low, mid-range and high values. The results are shown in Figure 1. As can be seen, when chitosan concentration is medium or high (*i.e. *>~0.4 mg/mL), by increasing the chitosan concentration, a peak in loading efficiency is observed which represent optimum value of pH (~ 5.1). Stirring oppositely charged polyelectrolytes in a solution causes their self-assembly due to the creation of strong but reversible electrostatic interactions. Many factors have been reported to affect the formation and stability of the polyelectrolyte complexes. Some examples include charge density and distribution on the polyelectrolytes, concentration and mixing ratio of the polymers, mixing order, molecular weight of the agents as well as the temperature and pH of the interaction environment (-). It is believed that cationic and anionic interaction sites are the main cause of streptokinase loading onto the chitosan. Therefore, at pH values between the isoelectric pH values of chitosan (*i.e*. ~6.0) and streptokinase (*i.e*. 4.7), the amino groups of chitosan are protonated and interact favorably with negatively charged carboxyl groups of streptokinase (2, 14, 29). Accordingly, at an optimum pH value (*i.e*. ~ 5.1, in this work), the most efficient interactions may be observed. Similarly, Alsarra *et al.* showed that when using electrostatic interactions between chitosan and TPP solution, pH and the ionic nature are of great importance in determining the loading efficiency. They also indicated that an optimum pH value is required to maximize the loading efficiency because of a proper ratio of the cationic and anionic interaction sites (14).

When chitosan concentration is low, the relation between pH value and loading efficiency follows a different pattern. Details show that herein, increase in pH would result in an increase in the loading efficiency. Apparently, the effect of streptokinase concentration on the loading efficiency masks the effect of pH, thus, variation in value of pH will not markedly affect the loading efficiency when the chitosan concentration is low (<~0.3 mg/mL). The reason for this finding (*i.e*. direct relation between pH value and loading efficiency) is complicated and not precisely clear. However, it may be explained as follows.

In the present work, as previously stated, since the preparation of nanoparticles was based on electrostatic interactions without any linker molecule, Polymer/enzyme charge ratio would be an important factor in the formation of nanoparticles. Streptokinase has a negative net charge when the solution pH is greater than 4.7. The protonated amino groups on the chitosan interact electrostatically with the negatively charged groups on the streptokinase. It is reasonable to assume that the alteration of ionizable state of the streptokinase promotes its interaction with amino groups of chitosan and leads to the high loading efficiency when pH value goes up while chitosan concentration is fixed at a low value. For instance Gan and Wang (7) used prepared BSA-loaded chitosan-TPP nanoparticles. They found that increase in mass ratio of chitosan to polyanion (TPP) leads to decrease in protein loading efficiency. This supports the idea that a smaller chitosan to TPP mass ratio is ideally appropriate to the protein loading during the formation of nanoparticles. One probable explanation is that a rise in the enzyme concentration will result in an intensified total negative charge carried by the long streptokinase molecules which consequently promotes electrostatic interactions between amino groups of chitosan and negatively charged streptokinase. Undoubtedly, further investigations are required for more in-depth clarifications about the underlying mechanism(s) and the conformational state of the chitosan/protein molecules present in the nanoparticles.

On the other hand, from the details, the increase in the chitosan level, in general, has a reverse and profound effect on the loading efficiency. As a matter of fact, the raise in environment viscosity with more chitosan level could be a main reason to the reduction of entrapment. This trend has already been reported (4, 30). It is also clear that the effect of chitosan concentration is pH dependent: while at low pH values, this effect is not considerable, when moving towards higher pH values, a substantial influence may be observed on the loading efficiency.

**Figure 2:**
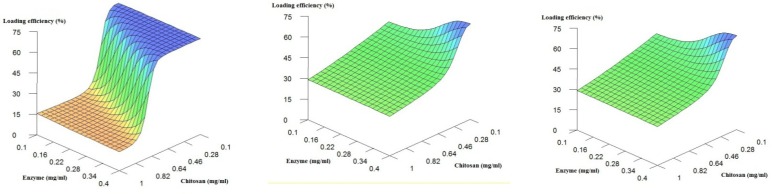
**.** 3D Plots of loading efficiency predicted by the ANNs model fixed at low, medium and high values of the buffer pH.

Figure 2 shows the effect of chitosan and enzyme concentration when the pH is fixed at low, medium and high values. It is obvious that in general, the decrease in the chitosan concentration leads to a considerable increase in the loading efficiency. As previously stated, the decrease in the concentration of the polymer in solution is major contributor to the decrease in solution viscosity. Less viscous chitosan solution results in more polymer chain mobility and less entanglement (20) which probably causes more efficient interactions between oppositely charged molecules. Additionally, from the Figure 2, the enzyme concentration does not appear to impose important influences on the loading efficiency. 

**Figure 3:**
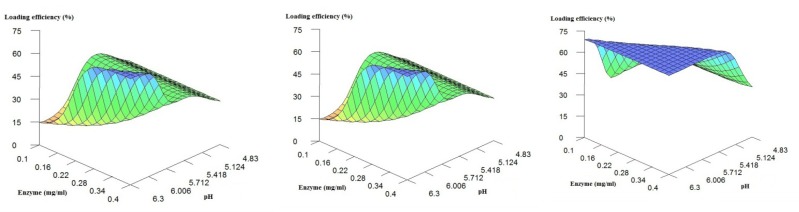
3D Plots of loading efficiency predicted by the ANNs model fixed at low, medium and high levels of the buffer pH.

The graphs in Figure 3 show the influence of enzyme concentration and pH effect on loading efficiency when chitosan concentration was fixed at low, medium and high concentrations. The results confirm the above findings:

- The increase in chitosan concentration in general results in decrease in loading efficiency.
- in respect to loading efficiency, there is an optimum pH (~ 5.1) when chitosan concentration is high or medium (*i.e*. > ~0.4 mg/mL)
- The increase in pH results in increase in loading efficiency when chitosan concentration is low.
- The change in enzyme concentration does not make considerable variations in the loading efficiency.

## Conclusion

In this study, we used ANNs to produce a model for examining the effect of chitosan concentration (mg/mL), buffer pH and enzyme concentration (mg/mL) on the loading efficiency (%) of streptokinase and formation of streptokinase-loaded chitosan nanoparticles. The 3D graphs used in this study showed that all the three factors evaluated have some effect on the enzyme loading efficiency. The level of enzyme did not appear to be dominant. On the contrary, high values of chitosan generally lead to a decrease in loading efficiency and in this case, pH could play an important role in the streptokinase loading efficiency, showing an optimum value ~5.1 for obtaining maximum efficiency. When the chitosan concentration reaches its minimum values, the loading efficiency is highest particularly when the pH is high too. 
